# Law Department of Niagara University

**Published:** 1887-06

**Authors:** E. C. Townsend

**Affiliations:** 73 White Building, Buffalo, N. Y.


### Editorial.

### *LAW DEPARTMENT OF NIAGARA UNIVERSITY.*

As announced some time since, Niagara University has added another department, that of law, to three already existing. The corps of professors, now complete, is as follows:

FACULTY.

Hon. Charles Daniels,
Dean and Professor of the Principles of Constitutional Law.

Hon. Charles Beckwith,
Professor of the Law of Equity Jurisprudence.

Hon. Albion W. Tourgee,
Professor of Legal Ethics.

LeRoy Parker,
Professor of Municipal Law, Contracts and Remedies.

Spencer Clinton,
Professor of the Law of Real and Personal Property.

James Fraser Gluck,
Professor of the Law of Corporations.

George Clinton,
Professor of Maritime Laws and Admiralty.

John G. Milburn,
Professor of the Theory of Law Codes and Codification.

Adelbert Moot,
Professor of the Law of Evidence

Tracy C. Becker,
Professor of Criminal Law and Procedure and Medical Jurisprudence

Charles P. Norton,
Registrar and Assistant to the Chair of Municipal Law, Contracts and Remedies.

E. Corning Townsend,
Assistant Instructor, Secretary and Treasurer.

The Law Department of Niagara University is, like its sister Medical Department, located at Buffalo, N. Y. It has been organized during the past year, and includes in its faculty and instructors some of the most prominent and able jurists of New York State. While it is under the direct management and supervision of the law faculty, it has, besides, a strong corps of special lecturers. The major part of these professors, instructors and special lecturers are lawyers of large practice, and the method of their instruction is an exposition of legal principles made practical by their own experience. The student thus learns the law in much the same way that all lawyers learn it in their practice.

The object of the department of law is to teach the theory and practice of common law, and it aims to afford such a training in its principles as will enable the student to practice his profession anywhere where the system of the common law prevails. Its training and method of instruction embrace the study of leading cases, lectures, recitations, drill in Moot Courts and theses written by the students on legal subjects.

Its course covers a period of two full years, beginning with the first week in October and ending the middle of May; with a post graduate or optional course of one year, to be pursued by students who have graduated or by students in special courses.

The prospectus soon to be published by the law department will show more fully the field of instruction contemplated.

The situation of the College at Buffalo is a peculiarly fortunate one During most of the college year four of the higher courts are sitting, in which the actual trial of important cases, often by distinguished counsel, is going on. During all of the college year special terms of the Supreme Court and the Superior Court of Buffalo are open, where judges sit daily to hear and determine questions touching almost every branch of the law. A term of the United States District Court is held in Buffalo in September of each year. The large and well-selected Bar Library will be free to students for consultation.

Places will be found for students in the offices of members of the Buffalo Bar, where it is expected they will spend their time when not actually engaged in attendance at the Law School Students will thus be afforded an opportunity of seeing, and to some extent assisting in, the actual details of law practice.

The admission of applicants who are candidates for a degree is regulated as follows: All graduates of literary colleges are admitted without examination. Candidates must be at least eighteen years of age and have received a good academic education They will be examined upon subjects prescribed by the faculty to be published in the announcement of the department. A certificate of the Board of Regents of New York State entitles a person to admission without examination. Applicants not candidates for a degree are also admitted without examination.

The degree conferred by the department on graduation is that of Bachelor of Laws The degree conferred at the end of the post graduate course is that of Master of Laws.

The tuition fee is one hundred dollars a year, payable one-half at the beginning and the other half at the middle of the year. The payment of the tuition fee shall be a pre-requisite to attendance. This fee admits the student to lectures and instruction in all the departments, and to the use of the law library and gymnasium.

Good board can be obtained in Buffalo at very reasonable prices.

For further inquiries, address the Secretary of the faculty,

E. C. TOWNSEND,

73 White Building, Buffalo, N. Y.

---

Dr. Edward Clark, who has for some months past been in New York with Dr. Kelsey studying diseases of the rectum, has returned to the city and will locate at 46 West Huron street, in the office formerly occupied by the late Dr. McNeil. Dr Clark will devote his attention exclusively to the practice of rectal surgery, and as we are acquainted with his high professional attainments we feel assured that he will rapidly acquire a lucrative practice in the specialty he has chosen.

